# Cognitive influencing factors of ICU nurses on enteral nutrition interruption: a mixed methods study

**DOI:** 10.1186/s12912-024-02098-2

**Published:** 2024-06-26

**Authors:** Huiling Pan, Chuanlai Zhang, Ruiqi Yang, Peng Tian, Jie Song, Zonghong Zhang

**Affiliations:** 1 Intensive Care Unit, The Second Affiliated Hospital of Chongqing Medical University, Yuzhong, Chongqing, People’s Republic of China; 2 School of Nursing, Chongqing Medical University, Yuzhong, Chongqing, People’s Republic of China

**Keywords:** Intensive care units, Nurses, Enteral nutrition, Enteral nutrition interruption, Attitude, Barriers, Mixed method

## Abstract

**Background:**

The incidence of clinically avoidable enteral nutrition interruptions is high. ICU nurses, as the implementers and monitors of enteral nutrition, have a close relationship between their cognitive level of enteral nutrition interruption and the incidence of enteral nutrition interruption. The level of ICU nurses’ cognition of enteral nutrition interruption and the key factors influencing the level of ICU nurses’ cognition of enteral nutrition interruption are not known.

**Objectives:**

This study aims to explore the cognitive level of ICU nurses on enteral nutrition interruption and delve into the key factors that affect their cognitive level from the perspective of management.

**Design:**

A sequential explanatory mixed methods research design was used.

**Methods:**

With the convenience sampling method, an online survey questionnaire was distributed to ICU nurses in Chongqing, and 336 valid questionnaires were collected. After the survey, ICU managers were invited to participate in qualitative interviews, in which 10 participants from five hospitals completed face-to-face individual semi-structured interviews and were analyzed with thematic analysis.

**Results:**

The survey found that ICU nurses had a good level of cognition towards enteral nutrition interruption but poor knowledge about the definition, causes, and consequences of enteral nutrition interruption, as well as negative attitudes toward active learning, assessment, and communication. And the longer work time in the ICU, joining the nutrition team, receiving systematic training, and acquiring relevant knowledge from academic journals more frequently were favorable to improving ICU nurses’ knowledge level of enteral nutrition interruption. Personal interviews further identified the key factors affecting their cognitive level, including (1) lack of knowledge, (2) lack of proactive thinking, (3) lack of enteral nutrition management programs, and (4) lack of quality management tools for enteral nutrition interruption.

**Conclusion:**

Although ICU nurses demonstrate a relatively high level of cognition, there is still room for improvement. ICU administrators must take specific measures to improve the knowledge of ICU nurses, especially in non-tertiary hospitals, in order to prevent nurse-induced enteral nutrition interruption in all ICUs and improve medical quality.

**Trial registration:**

Not applicable.

**Supplementary Information:**

The online version contains supplementary material available at 10.1186/s12912-024-02098-2.

## Introduction

Critically ill patients often experience physiological, pathological, and metabolic disorders that limit nutritional intake, and the prevalence of malnutrition is as high as 38–78% [1]. Malnutrition refers to a state of energy or nutrient deficiency caused by inadequate intake or utilization barriers [2], and it is a major factor contributing to adverse clinical outcomes for patients. Studies have found [3–5] that malnutrition in ICU patients increases the incidence of complications such as ICU length of stay, days of mechanical ventilation, infections and organ failure, and mortality. Therefore, nutritional therapy is particularly important in the management of critically ill patients.

Enteral nutrition (EN) has become the preferred nutritional support treatment for ICU patients due to its alignment with normal physiological metabolic processes [6]. Guidelines recommend [6, 7] that ICU patients should receive 80–100% of their target feeding volume within 3–7 days of initiating EN. 60–75% of patients in the ICU, however, as shown in several studies [8–10], do not reach the target feeding volume. Research [10] has found that the feeding deficiency rates were 54% and 15% (*p* < 0.001) on trial days with and without enteral nutrition interruption (ENI), respectively, indicating a positive correlation between ENI and insufficient feeding.

Enteral nutrition interruption (ENI) [11] is defined as an interruption of EN lasting 1 h or more with continuous enteral feeding or if the patient does not receive the expected amount of nutrients within 30 min with intermittent enteral feeding. Studies have found [10] that the average ENI time for ICU patients is up to 12 (6–24) hours per day. The causes of ENI are underestimated target feeding volumes, feeding intolerance, medical procedures, etc., which can be divided into patient factors and subjective factors [12, 13]. Among these, avoidable subjective factors related to medical operations account for approximately 72% of the total time of ENI [14, 15]. This is related to multiple factors such as physicians, nurses, frontline administrators, and healthcare institution management. ICU nurses, as the primary role in EN screening, assessment, implementation, monitoring, and complication intervention, are closely related to the occurrence of ENI in patients [16]. Studies have shown [17] that nurses not starting EN in a timely manner after medical procedures or outpatient examinations are the primary cause of ENI.

The Theory of Reasoned Action [18] proposes that individuals make behavioral decisions through rational thinking, and this decision-making process is influenced by various factors such as knowledge, attitude, and social environment. Thus, nurse-induced enteral nutrition interruption may be related to their level of knowledge, beliefs, and consequent practice behaviors related to ENI. To explore the current situation of ENI caused by ICU medical staff, previous studies [19] have examined the cognition of ENI among ICU medical staff in Wuhan. Little study, however, has been found to explore the key factors that affect their cognitive status. Currently, ICU managers lack a unified and standardized EN management plan. Furthermore, ICU nurses and doctors have different levels of knowledge, and nurses interact with patients more frequently, so a questionnaire is needed to evaluate ICU nurses’ cognition of ENI.

ICU manager [20] refers to the doctor or nurse who is responsible for the daily operation, management, supervision, and improvement of the ICU. ICU managers, as one of the key personnel in the whole link management and quality control of enteral nutrition, usually view problems from an overall perspective, and their perspectives and observations are more objective, in-depth, and comprehensive, which helps us understand the difficulties and challenges of ICU nurses in practice. We, therefore, use a sequential explanatory mixed methods research design [21] to investigate the cognitive level and influencing factors of ENI among ICU nurses through a cross-sectional survey. Based on the results, we will develop an interview outline to delve into the key factors influencing ICU nurses’ cognition of ENI from the perspective of ICU managers. This will lay the foundation for developing targeted interventions aimed at improving ICU nurses’ cognition of ENI, and provide the basis for improving the EN management program, so as to avoid nurse-induced ENI and improve medical quality.

## Methodology

### Research design

A sequential explanatory mixed methods research design [21] was used that included both quantitative and qualitative research. The interview guide for the qualitative research was developed based on the findings of the quantitative research and served to complement and explain the quantitative results.

### Quantitative research

#### Participants

Convenience sampling was used to conduct a cognitive survey on ENI among ICU nurses in Chongqing. The recruited object of this study was ICU registered nurses who had worked in general ICUs for at least one year. The first page of the questionnaire describes the purpose of this study and informed consent. Respondents can only access the survey questions after giving informed consent. After completing and submitting the survey, participants were considered to have given informed consent. In addition, researchers can judge according to the basic information filled in by participants to exclude those who do not meet the inclusion criteria. The sample size of this study was at least 193 according to previous similar studies [22].

#### Data collection

The scale used in this study is the “ICU Healthcare Providers’ ENI Knowledge, Attitude, and Practice Scale,” developed by the Yuanyuan Mi team in 2022 [22], which is used to understand the current level of knowledge, attitude, and practice of ENI among ICU medical staff. This scale comprised three dimensions: knowledge, belief, and practice, with 14, 10, and 17 items, respectively, and total score ranges of 14–70, 10–50, and 17–85. Items were rated using a Likert 5-point scale, with 1 indicating “not at all,” 2 “uncertain,” 3 “slightly,” 4 “fairly,” and 5 “completely.” Scores below 4 indicated poor cognitive levels of ENI among ICU nurses; scores equal to or greater than 4 indicated that ICU nurses have a good level of ENI awareness. Reportedly, the Cronbach’s alpha for the original scale was 0.953, the test-retest reliability was 0.795, and the total content validity coefficient was 0.975, indicating that the scale had good reliability and validity. In addition, the Cronbach’s alpha was 0.965 when the scale was retested using data from this study.

In this study, 10 demographic variables and the “ICU Healthcare Providers’ ENI Knowledge, Attitude, and Practice Scale” developed by the Yuanyuan Mi team [22] were converted into an online questionnaire. A cross-sectional survey was conducted among ICU nurses in Chongqing in October 2023. 366 questionnaires were distributed through the questionnaire star platform, and 366 were recovered, with a recovery rate of 100%. Two researchers checked the content of the questionnaire and the duration of the questionnaire, deleted 30 invalid questionnaires, and finally found 336 valid questionnaires, for an effective rate of 91.8%.

#### Data analysis

Data were downloaded from the Questionnaire Star platform and analyzed in SPSS 27.0. Statistical significance was set at *p* < 0.05. Means (standard deviations) and frequencies (percentages) were used for descriptive statistics. Differences and associations between ICU nurses’ EN cognition scores and demographic variables were analyzed using t-tests, chi-square tests, and binary logistic regression. Pearson’s correlation was used to assess the relationship between the total cognition score and the scores of each dimension.

### Qualitative research

#### Participants

Purposeful sampling was used to select ICU EN managers willing to participate in qualitative interviews from hospitals where the questionnaire was administered. Eligible participants included healthcare providers from general ICUs involved in EN management for at least three years and willing to participate in this semi-structured interview. A total of 10 ICU managers were included in this study for personal interviews. Information saturation [23] was reached at interview 8, meaning that no new themes emerged at the end of the interview process. Two further interviews were conducted to confirm the results.

#### Data collection

Data were collected through semi-structured interviews conducted by the first and second authors with participants in December 2023. The interview guide (see to S1) was developed by the lead author, guided by the Theory of Reasoned Action [18], and based on questionnaire results, a review of domestic and international literature, and expert consultation. Participants were contacted by phone before the interview to explain the purpose and significance of the study, obtain informed consent regarding confidentiality principles, recording, and other issues. Interviews were conducted at mutually agreed-upon times, ensuring privacy and a quiet environment. The interview time should be controlled at about 30 min. During the interviews, non-verbal cues such as body language, facial expressions, and tone of voice were observed and recorded along with audio recordings. A pilot interview was conducted with two ICU managers meeting the inclusion criteria before the qualitative study’s implementation, but their data were not included in the final analysis.

#### Data analysis

Audio recordings and written notes were transcribed verbatim within 24 h of the interview’s conclusion and stored on a computer for backup. Data analysis was based on the Theory of Reasoned Action [18] and aimed to identify key factors influencing the improvement of ICU nurses’ cognitive levels regarding ENI. A deductive thematic analysis approach [24] was employed, involving the following steps: (a) familiarization with the data; (b) initial code generation; (c) theme search based on initial codes; (d) theme review; (e) theme definition and labeling; and (f) report writing.

### Quality control

To ensure reliability, the research team met regularly, and team members reviewed the study data and analysis results. For the quantitative study, the online survey was anonymous. To ensure the authenticity and validity of the questionnaire results, each respondent was given only one chance to answer the questionnaire and was required to answer all the questions before submitting the questionnaire. To prevent the inclusion of low-quality questionnaires, it was assumed that each question would take no less than 2 s to answer, and in combination with the number of demographic characteristics entries (10) and scale entries (41), questionnaires with an answer time of less than 2 min were excluded from this study. The researcher observed and collected the filled-in data through the background of the questionnaire and double-checked the extracted information to ensure the completeness of the information. In the qualitative study, interview transcripts were collected by two research members trained in qualitative research, and one researcher organized the audio-recorded interviews into text within 24 h of the end of the interviews, which was then returned to the interviewees for confirmation by two researchers who repeatedly read and proofread the information. Participant recruitment, interviews, and data analysis were conducted simultaneously to help researchers determine information saturation. No repeat interviews were conducted.

### Ethical considerations

Ethical approval was obtained from the ethics committee of the Second Affiliated Hospital of Chongqing Medical University (Ke Lunshen No. (139) in 2023). The front page of the questionnaire sent to potential participants during the quantitative phase had an “informed consent” option, which was clicked on to allow participants to access the electronic questionnaire. Participants who submitted the questionnaire were considered to have obtained their informed consent. Participants in the quantitative phase volunteered their participation, and the questionnaire’s demographic data did not include names. Each participant was assigned a numerical code to ensure the confidentiality of survey responses. In the qualitative phase, participants provided written informed consent, and their interview recordings were analyzed anonymously and reported solely for research purposes by the study team.

## Results

### Quantitative phase

#### Demographic characteristics of ICU nurses

Among the 366 participants who completed the questionnaire, 336 (91.8%) were considered to have provided valid questionnaires. The mean age of the 336 study subjects was 31.24 ± 5.68 years, ranging from 22 to 59 years old. Among them, 192 (57.1%) nurse had junior professional title, a total of 285 (84.8%) held a bachelor’s degree or higher, and the average ICU working time was 6.88 ± 5.05 years. Most of the nurses worked in tertiary care hospitals [*N* = 212 (63.1%)], but a few were members of the nutrition team [*N* = 83 (24.7%)]. This survey showed that only 54 (16.1%) nurses had received systematic training on knowledge related to enteral nutrition, and only 25 (7.4%) nurses reported that they regularly obtained knowledge related to enteral nutrition from academic journals. (See Table 1)

**Table 1:** Demographic characteristics of ICU nurses(*N* = 336)

Variable	*n*(%)
**Gender**	
Male	31(9.2)
Female	305(90.8)
**Age**	
≤ 30 years old	187(55.7)
31 to 45 years old	137(44.8)
≧ 46 year old	12(3.6)
**Professional title**	
Junior title	192(57.1)
Intermediate title	120(35.7)
Senior title	24(7.1)
**Work time in ICU**	
≤ 5 years	154(45.8)
6 to 10 years	115(34.2)
>10 years	67(19.9)
**Academic qualifications**	
Below bachelor degree	51(15.2)
Bachelor’s degree or higher	285(84.8)
**Hospital grade**	
Grade III, Class A hospital	175(52.1)
Grade III, Class B hospital	37(11.0)
Others	124(36.9)
**Member of the Nutrition Team**	
Yes	83(24.7)
No	253(75.3)
**Training Related to Enteral Nutrition**	
Not studied before	24(7.1)
Studied but not comprehensive	258(76.8)
Systematically studied	54(16.1)
**Main learning pathways**	
School	4(1.2)
Hospital/Department Lectures	243(72.3)
Attending academic conferences outside	68(20.0)
Books and the internet	21(6.3)
**Frequency of acquiring knowledge about enteral nutrition from academic journals**	
Never	11(3.3)
Seldom	128(38.1)
Sometimes	167(49.7)
Often	25(7.4)
Always	5(1.5)

#### Cognitive level of ICU nurses regarding enteral nutrition interruption

As shown in Table 2, the mean score of ICU nurses’ knowledge of enteral nutrition interruption was 165.04 (22.86), which was higher than 164 (41 × 4), i.e., the cognitive level of ICU nurses regarding ENI was better. On the knowledge dimension, the mean score of ICU nurses’ knowledge of the definition, causes, and consequences of ENI was lower than 4, which was poor in this area; while " Unless contraindicated, the head of the bed should be elevated by 30–45° during EN administration to critically ill patients " and “When the medical and nursing-related examination, diagnosis, and treatment procedures are completed, enteral nutrition feeding should be resumed in a timely manner” had the highest scores, which were both higher than 4, indicating better knowledge in this area. The mean scores of ICU nurses in the belief dimension of ENI were all higher than 4, indicating better beliefs. On the behavioral dimension, ICU nurses scored higher than 4 on all behaviors except for lower scores on active learning about ENI, active patient assessment, and communication with physicians.

**Table 2:** Cognitive level total mean score (*n* = 336)

Cores	Mean(SD) Scores(*n* = 336)
Knowledge Dimension	51.5(9.62)
Belief Dimension	43.65(7.62)
Behavior Dimension	69.88(10.13)
Total Score	165.04(22.86)
K1. Under the premise of continuous feeding, the interruption of enteral nutrition lasting for 1 h or more can be defined as enteral nutrition interruption(ENI)	3.07(1.01)
K2. Under the premise of intermittent feeding, it can be defined as ENI if the patient does not receive the expected nutritional goals within 30 min after three infusions of 30 min each per day	2.97(1.09)
K3. ENI has the potential to significantly impact the patient’s energy goals, subsequently elevating the risk of nutritional deficiencies	3.47(0.99)
K4. ENI is positively correlated with the severity of the patient’s condition, hospitalization costs, and the achievement of target calories	3.42(0.95)
K5. Hemodynamic instability, elevated intra-abdominal pressure, and gastrointestinal-related complications, including intestinal obstruction, anastomotic leakage, and celiac disease, are significant contributing factors to ENI	3.63(0.90)
K6. Medical and nursing procedures related to examination, diagnosis, and treatment, including general anesthesia surgery, radiology examination, endotracheal fiberscope examination, establishment or replacement of artificial airways, position changes, sputum suction, and others, can serve as reasons for the ENI	3.72(0.90)
K7. Difficulties encountered during tube placement, tubing blockage, displacement, and dislocation of nutritional infusion tubes in critically ill patients are causes of feeding interruption	3.67(0.84)
K8. For critically ill patients admitted to the hospital, enteral nutrition (EN) should be promptly initiated within 24 to 48 h if both gastrointestinal function and hemodynamic stability are maintained.	3.99(0.89)
K9. In critically ill patients receiving EN via fractionated push and intermittent gravity drip, gastric residual volume (GRV) should be routinely monitored prior to each feeding. When continuous nutrition pump infusion is utilized, GRV monitoring should occur at least every 4 h to ensure patient safety	3.95(0.88)
K10. Sedative and analgesic medications can impact gastrointestinal motility, potentially leading to delayed gastric emptying. Dynamic assessment of patients’ pain levels and sedation depth is crucial, and minimization of sedative and analgesic use should be prioritized when clinically appropriate and aligned with patient preferences.	3.85(0.82)
K11. Gastrointestinal stimulant drugs can alleviate symptoms of gastrointestinal intolerance among critically ill patients	3.79(0.86)
K12. For critically ill patients exhibiting elevated intra-abdominal pressure (IAP > 12 mmHg), routine monitoring of IAP is essential. Adjustments to the rate and volume of EN should be made based on the individual’s IAP levels	3.49(0.99)
K13. Unless contraindicated, the head of the bed should be elevated by 30–45° during EN administration to critically ill patients	4.38(0.78)
K14. When the medical and nursing-related examination, diagnosis, and treatment procedures are completed, enteral nutrition feeding should be resumed in a timely manner	4.10(0.84)
A1. I think it is very important for ICU nurses to have knowledge about ENI	4.39(0.796)
A2. I believe that hospitals (or departments) should provide formal training on EN tolerance	4.35(0.82)
A3. I think that having more knowledge about ENI is very helpful to my clinical work	4.34(0.83)
A4. I think it is important to assess the nutritional status of all patients admitted to the ICU	4.38(0.81)
A5. I think that the assessment of nutritional status in ICU patients necessitates the collaborative involvement of both healthcare professionals and nurses.	4.42(0.80)
A6. I think it is important to choose the appropriate EN preparation/formulation to prevent ENI	4.35(0.82)
A7. I think it is important to establish and select the route of EN infusion	4.38(0.81)
A8. I think the management of EN feeding position is very important	4.39(0.81)
A9. I think it is important to develop a standardized EN management program to prevent and manage feeding interruptions	4.37(0.81)
A10. I think prevention of ENI is more important than treatment	4.29(0.86)
B1. I proactively seek knowledge regarding the ENI	3.42(0.81)
B2. I proactively communicate with patients or their families the importance of EN and inform them of the potential harms of its interruption	3.49(0.91)
B3. Upon admitting ICU patients, I promptly assess their nutritional status and communicate with the attending physician	3.68(0.85)
B4. Before initiating EN, I collaborate with the doctor to select and establish the correct feeding route for EN	3.71(0.89)
B5. I strictly adhere to hand hygiene protocols during the administration of EN	4.25(0.80)
B6. In the absence of medical contraindications, I elevate the head of the bed for patients receiving EN support to 30–45 degrees	4.53(0.71)
B7. During EN feeding, I monitor patients for symptoms of feeding intolerance, such as nausea, vomiting, reflux/aspiration, and abdominal distension, and promptly report any findings to the doctor	4.46(0.72)
B8. I would discontinue EN when patients require urgent airway establishment/replacement, such as intubation or tracheotomy	4.51(0.71)
B9. I would suspend EN during bedside X-ray imaging or bronchoscopy procedures.	4.34(0.85)
B10. In the event of a patient’s clinical condition deteriorating to the point of requiring immediate surgery or anticipated full anesthesia within 4–8 h, I discontinue EN	4.50(0.75)
B11. For patients exhibiting feeding intolerance, I investigate the underlying reasons and discuss with the doctor whether to discontinue EN	4.13(0.84)
B12. I would stop EN for patients whose shock cannot be corrected, whose hemodynamics and tissue perfusion targets (map < 50 mmHg), and whose hemodynamic stability can only be maintained by gradually increasing the dose of vasoactive drugs	4.0(0.94)
B13. I would discontinue EN in a patient with uncontrolled life-threatening hypoxemia, hypercapnia, or acidosis.	4.06(0.93)
B14. I would stop EN in critically ill patients with active upper gastrointestinal bleeding or intestinal ischemia	4.47(0.75)
B15. I would stop EN in patients with increased bladder pressure (IAP > 20 mmHg).	4.02(0.97)
B16. During EN support, if gastric residuals exceed 250 mL on two consecutive measurements, I alert the doctor to consider the use of gastrointestinal motility agents.	4.23(0.87)
B17. For patients with gastric feeding intolerance refractory to prokinetic agents or considered high risk for aspiration, I will communicate with the doctor to establish a post-pyloric feeding route	4.08(0.98)

#### Pearson’s correlation analysis among knowledge, belief, and behavior dimensions

As shown in Table 3, there was a strong positive correlation between the total cognitive score and the scores for the knowledge, belief, and behavior dimensions (*r* = 0.830, 0.766, and 0.850, respectively, *P* < 0.01). There was also a positive correlation between the knowledge dimension score and the scores for the belief and behavior dimensions (*r* = 0.487 and 0.549, respectively, *P* < 0.01). Furthermore, there was a positive correlation between the belief dimension score and the behavior dimension score (*r* = 0.535, *P* < 0.01).

**Table 3:** Pearson’s correlation analysis among knowledge, belief, and behavior dimensions

Dimension	Knowledge	Belief	Behavior	Total
Knowledge	1.000	0.487*	0.549*	0.830*
Belief	0.487*	1.000	0.535*	0.766*
Behavior	0.549*	0.535*	1.000	0.850*
Total	0.830*	0.766*	0.850*	1.000

#### Univariate analysis of knowledge, belief and behavior against demographic characteristics

ICU nurses were deemed to have a low cognitive capacity about ENI if they received a single-item score of less than 4. Therefore, a cutoff value of ≥ 4 was used to categorize the participants’ total cognitive scores, knowledge dimension scores, belief dimension scores, and behavior dimension scores into two categories: low (= 0) and high (= 1). These were used as dependent variables. Univariate analysis of ICU nurses’ demographics and cognitive scores showed that age, nutrition team membership, and frequency of acquiring relevant knowledge from academic journals were associated with ICU nurses’ level of cognition about ENI; professional title, nutrition team membership, systematic training, and frequency of acquiring relevant knowledge from academic journals were associated with ICU nurses’ knowledge scores about ENI; and frequency of acquiring relevant knowledge was associated with ICU nurses’ ENI belief dimension and behavioral dimension scores. A P-value of < 0.05 was considered statistically significant. (See Table 4)

**Table 4:** Univariate analysis of knowledge, belief and behavior against demographic characteristics (*N* = 336)

	Total score	Knowledge Score	Belief Score	Behavior Score
Gender	0.486*	0.518*	0.881*	0.307*
Age(Years)	0.035*	0.062*	0.258*	0.314*
Professional title	0.438*	0.038*	0.151*	0.623*
Work time in ICU(Years)	0.545*	0.083*	0.085*	0.488*
Academic qualifications	0.495*	0.675*	0.397*	0.556*
Hospital grade	0.650*	0.727*	0.164*	0.711*
Member of the Nutrition Team	0.021*	< 0.001*	0.107*	0.579*
Training Related to Enteral Nutrition	0.081*	< 0.001*	0.080*	0.422*
Main learning pathways	0.101*	0.094*	0.147*	0.438*
Frequency of acquiring knowledge about enteral nutrition from academic journals	< 0.001*	< 0.001*	0.012*	< 0.001*

*Note* * indicates the P-value

#### Factors associated with improving ICU nurses’ cognitive level

Variables with a P-value of < 0.10 from the univariate analysis were included as independent variables in a logistic regression model. The results showed that a high frequency of reading academic journals was a facilitating factor for improving ICU nurses’ cognitive level regarding ENI. Additionally, longer work time in the ICU, participation in nutritional groups, receipt of systematic training, and a high frequency of acquiring related knowledge about EN from academic journals were promoting factors for enhancing ICU nurses’ knowledge dimension scores regarding ENI (see Table 5).

**Table 5:** Multivariate analysis of Knowledge Dimension Score and demographic characteristics

Independent variable	β	SE	*P*	OR(95%CI)
**Work time in ICU**				
≤ 5 years old				1
6 to 10 years old	-0.728	0.295	0.014	0.483(0.271–0.861)
>10 years old	0.141	0.327	0.667	1.151(0.607–2.185)
**Member of** **the Nutrition Team**	-0.601	0.286	0.036	0.549(0.313–0.960)
**Training Related to Enteral Nutrition**				
Not studied before				1
Studied but not comprehensive	-0.578	0.479	0.228	0.561(0.219–1.435)
Systematically studied	0.301	0.555	0.588	1.351(0.455–4.013)
**Frequency of acquiring knowledge about enteral nutrition from academic journals**				
Never				1
Seldom	0.424	0.828	0.609	1.527(0.302–7.736)
Sometimes	0.794	0.824	0.335	2.213(0.440-11.133)
Often	2.724	0.964	0.005	15.235(2.301-100.855)
Always	2.401	1.422	0.091	11.034(0.680-178.973)
Constant	-0.321	0.938	0.732	0.725

### Qualitative phase

Ten ICU managers with bachelor’s degrees or above, ages ranging from 40 to 53, took part in individual semi-structured interviews from five hospitals. The duration of the interviews was roughly 12–36 min (see to S2). Four key factors were identified from qualitative data analysis that influence ICU nurses’ cognitive level regarding ENI: (1) Lack of knowledge; (2) Lack of active thinking; (3) Lack of EN management plans; and (4) Lack of quality management tools for ENI.

#### Lack of knowledge

According to participants, ENI is common in the ICU and is related to ICU nurses’ lack of knowledge about it. Many nurses are unclear about the definition, causes, and consequences of ENI. As Participant 5 described, ‘Many nurses are not yet aware of the concept of ENI and do not know how long a sustained pumping pause is an interruption of enteral nutrition, so much so that they are not particularly concerned about the time of restarting EN after a pause in EN, which leads to an increase in the duration and frequency of ENI in patients’. Furthermore, many participants stated that many nurses believe that pausing EN for a few hours during continuous enteral feeding does not constitute an interruption because the gastrointestinal tract remains active, which can damage a patient’s gastrointestinal function. Therefore, pausing for a few hours is similar to intermittent enteral feeding, allowing the patient’s intestine to rest. ICU nurses have a vague understanding of the definition and causes of ENI. What’s more, Participant 9 added, ‘Many nurses directly suspend EN when the gastric residual volume (GRV) exceeds 200 mL! Sometimes, when the GRV is assessed to be below 200 mL, the returned nutrient solution is discarded without realizing the relationship between ENI and adverse outcomes related to inadequate feeding’.

#### Lack of active thinking

Participants believed that the limitations in ICU nurses’ cognitive level regarding ENI were related to their mechanical work and lack of active thinking. Various reasons for ICU nurses’ lack of active thinking were described. Notably, due to limited human resources, ICU nurses, apart from handling doctor’s orders and basic care, also need to deal with emergencies and adverse reactions among critically ill patients, such as resuscitation, vomiting, and diarrhea. At the same time, they need to dynamically assess patients and fill out numerous assessment forms, making their workload heavy. As Participant 5 explained, ‘For example, when ICU nurses administer a doctor’s order of 1000 mL of nutrient solution to a patient, they routinely adjust the feeding speed, mechanically fill out various forms, and habitually assess the patient’s enteral feeding intolerance. If the patient tolerates it, they simply finish the feeding and move on, rarely thinking about whether the patient’s EN feeding has reached their nutritional goals……If the patient is intolerant, they habitually discard the syringe return fluid when the GRV is greater than 200 mL or even 50 mL and directly suspend the patient’s EN!’ Participants felt that ICU nurses, as implementers and monitors of EN, had a diminished sense of active learning as their sense of active thinking weakened. Participant 6 stated, ‘ICU nurses lack knowledge of biochemical indicators related to EN (such as phosphorus), hemodynamics, patients’ total enteral nutrition target, calories, and protein, and believe that nurses do not need to master these, lacking active learning consciousness’. Although many hospitals have EN management teams, most participants stated that team members are not very motivated, often forced to accept tasks, and lack active learning consciousness, which may be related to their lack of demand, competition, and conflict of interest.

#### Lack of EN management plans

It was evident from the interviews that the management level varies among different medical units, and there is inconsistency in the quality of care provided by doctors and nurses. The absence of standardized EN management plans that can be referred to has limited the improvement of ICU nurses’ cognitive level regarding ENI. For example, there is a lack of solutions to address inconsistencies between theory and practice. Participant 4 described, ‘Nurses are confused about the different gastric residual volume thresholds recommended by multiple guidelines, resulting in behaviors such as suspending EN when the volume exceeds 200mL. There is a lack of regulations regarding GRV thresholds and guidance on how to adjust or reduce the feeding rate in our department’. Participant 1 stated, ‘Nurses are unclear about whether it is necessary to routinely aspirate gastric residuals every 4–6 hours’. Participant 6 added, ‘The department lacks an active feeding strategy for restarting enteral nutrition to promote early active venting of patients’. Furthermore, participants felt that the management of EN in ICU patients requires multidisciplinary collaborative management, but the triad of physicians, nurses, and nutritionists each had their own role and lacked a closely linked management process. Participant 7 described, ‘ICU doctors have better knowledge of nutrition, less consultation with the Nutrition Department is requested, and nutritionists are unable to dynamically assess the EN status of patients in a timely manner, to the extent that it is mostly left to the ICU doctors themselves to determine the problem of patients’ EN compliance’. And participant 3 said, ‘Currently, ICU nurses put a lot of effort into screening, assessment, implementation, monitoring, and complication intervention of EN, and their awareness is gradually increasing (smiled), while physicians are less involved in the management of the EN process!’ What’s more, participants described that the initial nutritional screening assessor varies from ICU to ICU, that some are nurses whereas others are physicians, that it is not yet known who leads the management of EN in ICU patients, and that there is a lack of a collaborative management process between the medical and nursing professions.

#### Lack of quality management tools for enteral nutrition interruptions

Participants noted that current clinical EN management primarily consists of EN guidelines, implementation procedures, nutritional screening tools, enteral nutrition tolerance assessment forms, and aspiration risk assessment forms. However, there is still a lack of quality management tools specifically designed for ENI. This makes it difficult for ICU nurses to identify avoidable causes of ENIs, which in turn hinders their ability to reduce the occurrence of such interruptions. Participants described some avoidable issues related to ENIs. Participant 6 described, ‘ICU nurses often pause EN when the amount of GRV exceeds 200 mL, lacking a standardized deceleration or reduction in volume’. Participant 2 described, ‘Clinical situations often arise where infusions are not completed within 24 hours……This is attributed to unreasonable infusion speed settings, excessive preoperative fasting durations, forgetting to report to doctors after suspensions, forgetting to restart infusions, and equipment malfunctions.” Although the EN management team has identified issues related to ENIs during the management process, they lack plans for implementation and problem-solving. They expressed a desire to use quality management tools to manage ENIs and reduce those caused by human factors.

## Discussion

Understanding the cognitive level and influencing factors of ICU nurses regarding ENIs is crucial, as their cognition has a direct relationship with achieving the nutritional targets for ICU patients’ EN [16]. This study helps ICU managers understand the key factors affecting the cognitive level of ICU nurses’ ENI in order to lay the foundation for ICU managers to develop targeted interventions aimed at improving the cognitive level of ICU nurses’ ENI. Analysis of the questionnaire revealed that ICU nurses generally have a good level of cognition regarding ENIs, with a poorer understanding of their definitions, causes, and consequences. Additionally, they exhibited a negative attitude towards actively seeking knowledge, assessing, and communicating. However, there is still room for improvement, such as by joining nutrition groups, receiving systematic training on EN, participating in related academic conferences, and regularly acquiring EN knowledge from academic journals. Based on this, ICU managers further explained the key factors influencing nurses’ cognitive levels: a lack of knowledge regarding ENIs, inactive thinking about achieving EN feeding targets, a lack of management processes for addressing inconsistencies between theory and practice, and a lack of quality management tools for ENIs. These findings provide a basis for ICU managers to improve EN management plans. Therefore, it is recommended that ICU managers accordingly develop targeted interventions aimed at improving ICU nurses’ cognition of enteral nutrition interruptions in order to avoid nurse-induced ENI and improve medical quality.

This study is consistent with the findings of Mi Yuanyuan [19] et al. that ICU nurses have a better level of ENI cognition. However, this study also found that the number of years working in the ICU and nutrition team members were the influencing factors for the ICU nurses’ ENI knowledge dimension scores. This may be related to the fact that only ICU healthcare workers in tertiary hospitals were included in the study by Mi Yuanyuan [19] et al. or to the fact that nutrition team members accounted for as much as one-third of the ICU nurses in the study by Mi Yuanyuan ^[19]^ et al. This is also a side effect of the unequal levels of ENI awareness among ICU nurses in different levels of hospitals. In the future, more ICU nurses in secondary hospitals can be included to explore the current status of ENI cognitive level of ICU nurses in different grades of hospitals. Furthermore, unlike previous studies ^[19]^, this study conducted qualitative interviews with ICU managers on the basis of a questionnaire survey of ICU nurses, which explored the key factors affecting the cognitive level of ICU nurses’ ENI in more depth and laid the foundation for ICU managers to formulate targeted interventions aiming to enhance the cognitive level of ICU nurses’ enteral nutrition interruption.

In this study, we found that high years of working experience in ICU, joining the nutrition team, receiving systematic training, and a high frequency of acquiring knowledge related to enteral nutrition from academic journals were the contributing factors to increasing the level of ICU nurses’ knowledge of enteral nutrition interruption. The longer the working years, the richer the clinical experience and related knowledge of ICU nurses. However, as shown in this study, nearly half [*N* = 154 (45.8%)] of the ICU nurses had less than 5 years of working experience; therefore, there is an urgent need to improve the level of ICU nurses’ cognition of ENI in other ways in order to balance the level of cognition of ICU nurses with different years of working experience. For example, by joining a nutrition team and receiving relevant systematic training, ICU nurses can be helped to gain a systematic, comprehensive, and in-depth understanding of knowledge related to enteral nutrition and to increase nurses’ awareness of and interest in the interruption of enteral nutrition [25]. This is to promote proactive thinking by ICU nurses and to improve their scores in proactive learning about interruption of enteral nutrition, proactive assessment of patients, and communication with physicians [26]. Further, ICU nurses can also compensate for knowledge blindness by frequently acquiring knowledge related to enteral nutrition from academic journals. Academic journals, as authoritative repositories of academic knowledge, have the most cutting-edge knowledge in the field, such as clinical guidelines and original research with practical guidance, and ICU nurses’ frequent acquisition of enteral nutrition-related knowledge from academic journals is conducive to a systematic and in-depth understanding of the guidelines, consensus, original research, and the frontiers of enteral nutrition in order to enhance nurses’ knowledge of enteral nutrition interruption. Therefore, ICU administrators can encourage nurses to join nutrition teams and conduct multi-pathway training to promote nurses’ acquisition of knowledge from academic journals in order to improve ICU nurses’ level of knowledge about enteral nutrition interruptions, as well as to promote nurses’ proactive thinking in order to avoid unnecessary enteral nutrition interruptions.

Nurses are susceptible to the influence of external factors, and procedures and systems are fundamental to regulating nurses’ behavior. The development of enteral nutrition management protocols is beneficial to standardizing ICU nurses’ management of patients with enteral nutritional feedings in order to improve the level of ICU nurses’ perception of enteral nutritional interruption. A national survey [27] found that enteral nutrition is usually prioritized lower than other urgent care needs for ICU patients. Furthermore, there is a lack of uniform and standardized clinical protocols for enteral nutrition management in critically ill patients [28, 29]. This has hindered the improvement of the level of ENI awareness among ICU nurses in different levels of hospitals to a certain extent and is not conducive to the homogenization of ICU healthcare personnel in various healthcare institutions. Enteral nutrition is critical to the recovery of ICU patients [4]. It is necessary to enhance ICU nurses’ knowledge of enteral nutrition management to facilitate the development of standardized enteral nutrition protocols [30, 31]. Currently, the threshold for GRV is not uniform in clinical settings, with 200–500 mL being the most common [32, 33]. This is not conducive to ICU nurses’ judgment of GRV thresholds, which may lead to some degree to nurse-induced ENI. Furthermore, guidelines have recommended that routine monitoring of GRV [7] during the EN may not be necessary, but most clinical nurses still habitually aspirate gastric residual to monitor patients’ gastrointestinal intolerance, which may be related to the ICU nurses’ fear of the risk of patients’ vomiting or aspiration [34] or to their insufficiently in-depth view of the problem. At the same time, there is currently a clinical controversy over whether the gastric residual aspirates should be returned or discarded [35]. This may explain, in part, why some ICU nurses currently choose to discard the gastric residual aspirates directly to avoid contamination, and some ICU nurses choose to tie back the gastric residual aspirates to minimize the risk of fluid and electrolyte imbalance in the patient. Therefore, there is an urgent need for the development of standard enteral nutrition management protocols to address the currently controversial issues and to standardize ICU nurses’ behavior regarding enteral nutrition management.

The formulation of the scheme is conducive to standardizing the behavior of nurses, but the optimization of the implementation effect of the scheme requires the application of quality management tools. Currently, there is a lack of quality management tools in clinical practice to monitor the rate of implementation of EN measures [5, 6]. Previous studies have shown [12, 13] that the reasons for ENI in ICU patients include hemodynamic instability, high GRV, and medical procedures. It is difficult to avoid ENI, but as shown by Kagan et al. [36], the use of nutritional management feeding platforms (such as the smART + platform) can monitor ICU patients’ ENI in real-time, calculate the amount of compensation needed when restarting, and ultimately help patients reach their EN goal. In other words, most ENIs caused by ICU nurses can be avoided through the use of management tools28. As a fine and process management method, the Plan-Do-Check-Act (PDCA) cycle method is a continuous quality management tool that targets clinical weaknesses, proposes countermeasures, and improves the implementation rate of measures. It has been widely used in ICU quality management [37]. Therefore, in the future, ICU managers can use quality management tools to dig deeper into the reasons for enteral nutrition interruption, promote the development and implementation of related plans, and solve the problem at the source in order to reduce avoidable enteral nutrition interruption, standardize nurses’ behaviors, and maximize the application of enteral nutrition management programs.

## Strengths and limitations

This study boasts both strengths and limitations. Leveraging the advantages of mixed methods research, we delved into the key factors influencing ICU nurses’ cognition of ENI from both the nurses’ and management’s perspectives. This lays the foundation for targeted interventions aimed at enhancing ICU nurses’ understanding of ENI, ultimately aiming to prevent such interruptions caused by the nurses themselves. Rather, we must acknowledge its limitations. Our use of sequential explanatory mixed methods means our ability to explore the critical factors influencing ICU nurses’ cognition of ENI is somewhat limited, but this could be addressed through alternative mixed methods designs. Furthermore, our study sample was limited to a geographical region, potentially limiting the generalizability of our findings. Future research could expand the scope of the investigation. Nevertheless, this study provides novel insights and valuable perspectives for ICU managers to improve their department’s EN management strategies.

## Conclusion

Overall, the level of ICU nurses’ cognition of enteral nutrition interruption is good, but there is still room for improvement. ICU nurses can improve the level of knowledge related to ENI and increase their proactive thinking about the management of enteral nutrition target feeding compliance by joining the nutrition team, participating in the systematic training of knowledge related to enteral nutrition, and frequently acquiring knowledge from academic journals. Furthermore, ICU managers should apply a quality management tool for enteral nutrition interruptions and develop targeted interventions aimed at improving ICU nurses’ cognition of enteral nutrition interruptions in order to provide a basis for improving the department’s enteral nutrition management program, so as to avoid nurse-induced ENI and improve medical quality.

### Electronic supplementary material

Below is the link to the electronic supplementary material.

Supplementary Material 1

Supplementary Material 2

## Data Availability

All data generated or analyzed during the study are available from the corresponding author [Chuanlai Zhang] on request.

## Abbreviations

ICUs: Intensive care units
EN: Enteral nutrition
ENI: Enteral nutrition interruption
GRV: Gastric residual volume
