# Supplementary material for: Cognitive influencing factors of ICU nurses on enteral nutrition interruption: a mixed methods study
Source: BMC Nurs. 2024 Jun 26;23:433. doi: 10.1186/s12912-024-02098-2 (PMC11201787; doi:10.1186/s12912-024-02098-2)
Supplement: Supplementary file 2 — Supplementary Material 2 [file 12912_2024_2098_MOESM2_ESM.docx]

| 1.Could you describe in as much detail as possible the process by which nurses in the ICU provided enteral nutrition support treatment to a patient? |
| --- |
| 2.Could you share your thoughts on the role of ICU nurses in the management of enteral nutrition and explore their relationship with enteral nutrition interruption? Why？ |
| 3.Is ICU nurses' knowledge of enteral nutrition interruption something you view as important/not important? Why? Please provide some examples. |
| 4.What do you think is the reason that affects ICU nurses' cognition level of enteral nutrition interruption? Why? How? |
| 5.How easy or hard is it for you to avoid the interruption of enteral nutrition as much as possible? Why? |
| 6.What are the advantages and disadvantages of enteral nutrition management for critically ill patients? Any suggestions? |

**S1**. Interview Questions

| ID | Age | Gender | Degree | Position | Professional Title | Work time in ICU(Years) | Interview Duration (min) |
| --- | --- | --- | --- | --- | --- | --- | --- |
| 1 | 35 | Female | Bachelor | Head nurse | high | 12 | 12:23 |
| 2 | 41 | Female | Bachelor | Head nurse | high | 20 | 25:16 |
| 3 | 41 | Female | Bachelor | Head nurse | high | 21 | 16:08 |
| 4 | 50 | Female | Bachelor | Head nurse | vice-senior | 30 | 21:39 |
| 5 | 36 | Female | Bachelor | Head nurse | middle | 11 | 36:15 |
| 6 | 52 | Male | Master | Associate chief  physician | vice-senior | 28 | 12:03 |
| 7 | 40 | Female | Doctoral | Associate chief physician | vice-senior | 12 | 12:02 |
| 8 | 49 | Female | Bachelor | Head nurse | high | 25 | 12:19 |
| 9 | 53 | Male | Doctoral | Chief physician | high | 30 | 12:25 |
| 10 | 49 | Female | Bachelor | Head nurse | high | 14 | 23:24 |

**S2**. Basic Information of ICU Managers
